# Persistent isokinetic knee flexion strength deficits at the time of return to sport are not associated with a second ACL injury

**DOI:** 10.1002/ksa.12718

**Published:** 2025-06-08

**Authors:** Axel Sundberg, Rebecca Hamrin Senorski, Johan Högberg, Ramana Piussi, Kristian Samuelsson, Roland Thomeé, Eric Hamrin Senorski

**Affiliations:** ^1^ Capio Ortho Center Gothenburg Sweden; ^2^ Sahlgrenska Sports Medicine Center (SSMC) Gothenburg Sweden; ^3^ Department of Health and Rehabilitation, Unit of Physiotherapy, Institute of Neuroscience and Physiology, Sahlgrenska Academy University of Gothenburg Gothenburg Sweden; ^4^ Sportrehab Sports Medicine Clinic Gothenburg Sweden; ^5^ Department of Orthopaedics, Institute of Clinical Sciences, Sahlgrenska Academy University of Gothenburg Gothenburg Sweden; ^6^ Swedish Olympic Committee Stockholm Sweden

**Keywords:** ACL reconstruction, hamstring strength, limb symmetry index, second ACL injury

## Abstract

**Purpose:**

To investigate the rate of a second anterior cruciate ligament (ACL) injury based on different levels of knee flexion strength limb symmetry index (LSI) at the time of return to sport (RTS) after ACL reconstruction with hamstring tendon autograft.

**Methods:**

Data was extracted from a rehabilitation registry for patients aged 15–40 years, who participated in knee‐strenuous sports pre‐injury (Tegner ≥ 6) and underwent ACL reconstruction with hamstring tendon autograft. Isokinetic knee flexion strength was analysed and reported as LSI. Patients were categorised into three groups (≥90%, 80%–89.9% and <80%) based on their LSI at reported time of RTS. Patients were followed for 2 years after ACL reconstruction to record a second ACL injury, and hazard ratios (HR) were calculated using a Cox proportional hazards model.

**Results:**

A total of 526 patients (48% female, mean age 22 ± 6) were included, with 51 (9.7%) second ACL injuries recorded within 2 years after ACL reconstruction. Among patients with LSI ≥ 90% (71%), 43 second ACL injuries (11.0%) occurred. The LSI 80%–89.9% group had 4 second ACL injuries (4.0%), and the LSI < 80% group had four injuries (8.2%). Persistent knee flexion strength asymmetry did not significantly influence the hazard of a second ACL injury. The LSI 80%–89.9% group had a lower hazard (HR 0.34, confidence interval [CI]: 0.12–0.94), while the LSI < 80% group showed no significant difference (HR 0.70, CI: 0.25–1.97) compared with the LSI ≥ 90% group.

**Conclusion:**

Persistent isokinetic concentric knee flexion strength asymmetry at RTS were not associated with a second ACL injury.

**Level of Evidence:**

Level III.

**Abbreviations**
ACL: anterior cruciate ligament
ACLR: anterior cruciate ligament reconstruction
ACL‐RSI: ACL‐return to sport after injury
BMI: body mass index
CI: confidence interval
HR: hazard ratio
HT: hamstring tendon
ICC: intra class correlation
KOOS: knee injury and osteoarthritis outcome score
K‐SES: Knee Self‐Efficacy Scale
LSI: limb symmetry index
PRO: patient reported outcomes
RTS: return to sport
ST: semitendinosus tendon

## INTRODUCTION

Athletes who have undergone anterior cruciate ligament (ACL) reconstruction face a significantly increased risk of a second ACL injury compared to the risk of a primary ACL injury. Up to 1 in 4 athletes under the age of 25 who have undergone ACL reconstruction sustain a second ACL injury, with the highest risk within the first 2 years after ACL reconstruction [54]. The choice of graft for ACL reconstruction remains a topic of debate, particularly regarding the graft's influence on the likelihood of a subsequent ACL injury [8, 28, 53]. The hamstring tendon (HT) autograft, a multi‐folded semitendinosus tendon (ST), at times supplemented with the gracilis tendon for a sufficient graft diameter [46], has increased in popularity in recent decades and is the most frequently used autograft for primary ACL reconstruction in Sweden [21, 37]. Data from Cristiani et al. [12] indicate that ACL reconstruction with an HT autograft results in greater anterior tibial translation compared to a bone‐patellar tendon‐bone autograft at six months. Patients with a HT autograft were reported to have a higher incidence of a persistent side‐to‐side difference of ≥5 mm, suggesting that the HT autograft may lead to compromised knee laxity. The hamstring acts as a synergist to the ACL by counteracting anterior translation of the tibia and controlling rotational stability [31]. Consequently, the importance of thorough rehabilitation of hamstring function following HT autograft has been emphasised. The regeneration of the ST after graft harvesting varies considerably, typically showing some level of regeneration within one year post ACL reconstruction [13]. Nevertheless, ST regeneration has been associated with an altered anatomy, including, but not limited to, the position of insertion, proximal retraction of the musculotendinous junction, and persistent reduction in physiological cross‐sectional area, which may affect hamstring force potential and knee flexion strength [24, 35].

Rehabilitation practitioners are advised to assess multiple aspects of muscle function after ACL reconstruction. Test batteries which consist of knee muscle strength and hop performance are commonly employed to inform return to sport (RTS) decision [30]. The limb symmetry index (LSI) is commonly used to interpret results from the muscle function tests, and as a part of the decision‐making process prior to RTS [18], where patients are recommended to pass given LSI cut‐offs. Whether a specific LSI cut‐off is relevant to predict the risk of a second ACL injury and what is the most appropriate cut‐off, remains unknown [48, 51]. To date, the most frequently advocated LSI cut‐off to clear patients for RTS is LSI ≥ 90% [9]. While quadriceps strength deficits prior to RTS have been linked to an increased risk of a second ACL injury [19, 29], the significance of persistent deficits in hamstring strength remains uncertain. Despite concerns about persistent deficits in knee flexion strength at the time of RTS [2, 26], there is limited evidence for knee flexion strength symmetry in protecting against a subsequent ACL injury [23]. The purpose of this study was to assess the rate of second ACL injury based on different levels of knee flexion strength LSI at the time of return to sport after ACL reconstruction. The hypothesis was that patients with lower hamstring strength symmetry would have a higher risk of a second ACL injury.

## METHODS

### Study design

This observational cohort study was written in accordance with the REporting of studies Conducted using Observational Routinely‐collected Data (RECORD) guidelines [5].

### Setting

The study was performed based on data from a rehabilitation outcome registry, Project ACL, located in Gothenburg, Sweden. Project ACL started in 2014 and aims to improve care for patients who sustain an ACL injury, through regular evaluation of muscle function during the rehabilitation process. Patients who sustain an ACL injury treated with or without reconstruction are eligible to register in Project ACL. Repeated evaluations in Project ACL are conducted according to a standardised protocol including tests of isokinetic strength of knee extension and knee flexion, hop performance, and validated patient‐reported outcomes (PROs). All tests are supervised by physical therapists trained in this standardised test procedure. Tests are performed at a pre‐set timeline of 10 weeks, 4, 8, 12 and 18 months, 2 and 5 years and then every fifth year after ACL injury or reconstruction as baseline. Upon registration in the project, patients are requested to provide baseline data, such as age, time from ACL injury to reconstruction, injury mechanism and anthropometrics. At the first test after ACL reconstruction the test supervisor adds information about the autograft used for reconstruction, Beighton score for hypermobility and can add information about additional surgical measures. It is important to note that Project ACL does not include details on the specific rehabilitation protocols performed by patients. The registry provides clinicians with objective data on evaluation of muscle function and patient‐reported outcomes but does not dictate rehabilitation progression or offer specific recommendations regarding rehabilitation strategies. Participation in Project ACL is voluntary, and informed consent is required prior to inclusion. Withdrawal from the registry can be made at any time, and participation does not affect the treatment of the individual patient. Ethical approval has been obtained from the Swedish Ethical Review Authority, with registration number: 2020‐02501.

### Participants

Patients eligible for inclusion were 15–40 years at ACL reconstruction, had a pre‐injury Tegner Activity Scale (Tegner) [47] score of ≥6, underwent primary ACL reconstruction with ipsilateral HT autograft, and attended Project ACL follow‐up with strength tests for knee flexion and extension. Additionally, all patients had RTS with a Tegner score of ≥6 at follow‐up, and a minimum of 2 years had passed since ACL reconstruction. Patients were excluded from the study if they reported RTS before 4 months or later than 18 months after ACL reconstruction and if they had not performed strength tests at the same follow‐up as when RTS was reported.

### Data source/measurements

Patient demographics (age, sex and Tegner level), ACL reconstruction date, occurrence of second ACL injury and reported time of RTS (Tegner ≥6) were extracted from Project ACL inception to January 2023. Isokinetic strength and hop test results were extracted from the follow‐up when patients reported RTS (4, 8, 12 or 18 months), indicating exposure to risk situations for a second ACL injury. Patients were categorised into three groups based on LSI of knee flexion strength: ≥90%, 80%–89.9% and <80%. A second ACL injury was registered in Project ACL by the patient or clinician and had to be clinically confirmed and recorded in the registry. However, there were no specific criteria required for verification, such as magnetic resonance imaging or arthroscopy.

### Strength and hop test procedure

Before the strength and hop tests, all patients were cleared by the supervisor according to the following criteria: minimal knee pain and effusion. Patients completed a test battery which consisted of isokinetic strength tests and three single leg hop tests. A warm‐up of 10 min on a stationary bicycle and practice trials of each test were performed before the test started. Isokinetic concentric strength tests of knee extension and flexion were performed at an angular velocity of 90° per second with a Biodex System 4 dynamometer (Shirley, NY, USA) (24). Three to five maximum effort repetitions were performed with 40 s of rest in between each repetition. Peak torque results were recorded in Newton metre (Nm) in the Project ACL database. Hop tests were conducted immediately after the strength tests, but only if the patients were already familiar with hop training during rehabilitation and the specific test procedure. The three single‐leg hop tests included were: vertical hop, hop for distance, and 30‐s side hop [20]. All hop tests were performed with both hands placed behind the patient's back. For the vertical hop, flight time was converted to centimeters (cm) with the Muscle Lab (Ergotest Technology, Oslo, Norway). Hop for distance was measured in cm, from toe at take‐off to heel at landing. Patients were required to perform a stable landing, that is, without movement of the landing foot, without assisting balance with the opposite foot, and without letting go of hands behind their back. Three to five repetitions of each test were allowed, and the best result for each leg in cm was recorded. For the 30‐s side hop test, patients performed one maximum effort trial by hopping as many times as possible over two lines 40 cm apart, and the number of hops without touching the lines were recorded. Results from hop tests were registered in the Project ACL database.

### Patient reported outcomes

The only PRO used in the present study was the Tegner scale which grades the level of knee demanding physical activity associated with the patient's sports participation. The Tegner scale ranges from 0 to 10, where level 0 represents sick leave, level 6 corresponds to sports such as badminton and volleyball, and 10 indicates elite level sports such as football or rugby. Project ACL uses a modified Tegner scale [3] starting from level 1, which represents walking on stable ground. In this study, a pre‐injury Tegner score of ≥6 was selected as it marks the threshold for knee‐demanding activities, potentially increasing the risk of a second ACL injury due to the higher knee‐demanding intensity and complexity [45]. The Tegner scale has reported good test‐retest reliability, with an intraclass correlation coefficient of 0.8 [7].

### Variables

The primary outcome of interest was the rate of a second ACL injury to either the ACL reconstructed knee or the contralateral knee, within the first 2 years after ACL reconstruction, in three different patient groups based on LSI in knee flexion strength (≥90%, 80%–89.9% and <80%). The group with LSI ≥ 90% served as the reference group per clinical guidelines for RTS following ACL reconstruction. Additionally, the hazard for a second ACL injury was examined between the LSI 80%–89.9% and LSI < 80% groups, with the former as the reference, under the assumption that a higher LSI would reduce the likelihood of a second ACL injury. Furthermore, a secondary analysis was conducted to explore a potential protective effect by evaluating patients who had achieved LSI ≥ 90% in both knee extension and single leg hop tests at the same test occasion as their knee flexion LSI.

### Statistical methods

Statistical analyses were performed using the Statistical Analysis System (SAS) software version 9.0 (Institute Inc., Cary, NC, USA). Patient demographics were presented as counts and percentages for categorical variables, and as means with standard deviations for continuous variables. Demographic variables were compared between patients who sustained a second ACL injury and those who did not using Fisher's exact test, chosen for its precision with small sample sizes and categorical data. Effect sizes were calculated as eta squared (*η*²) for continuous variables (ANOVA) and Cramér's V for categorical variables (chi‐square test), with higher values indicating stronger associations or greater variance explained. For the primary analysis, a univariable Cox proportional hazard model was used to investigate the association between the LSI of knee flexion strength, differentiated into three groups (LSI ≥ 90%, LSI 80%–89.9%, and LSI < 80%) and the hazard to experience a second ACL injury. In this model, a second ACL injury (yes/no) served as a dependent variable. A hazard ratio greater than 1 suggests an increased hazard for a second ACL injury, while a hazard ratio below 1 implies a decreased hazard for a second ACL injury. Hazard ratios for a second ACL injury were reported with a 95% confidence interval (CI). Statistical significance was defined as a *p*‐value < 0.05. For the secondary analysis, a multivariable Cox proportional hazard model with a 95% CI was performed accounting for concurrent symmetry in knee extension strength and single leg hop performance for the three different knee flexion strength LSI groups (LSI ≥ 90%, LSI 80%–89.9%, and LSI < 80%). Knee extension strength and hop performance LSI ≥ 90% were dichotomised to yes/no. The occurrence of a second ACL injury was illustrated with a Kaplan–Meier curve, and differences between groups were tested using the log‐rank test.

## RESULTS

**Figure 1:**
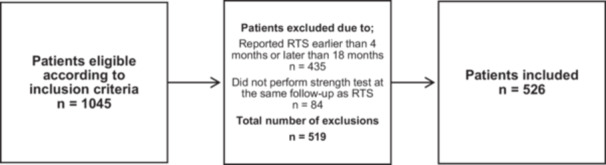
Flow‐chart of the patient inclusion and exclusion process. RTS, return to sport.

Out of the 1045 patients eligible according to the inclusion criteria from the Project ACL database, a total of 526 patients were included in this study (Figure 1). Among the 526 patients, 51 second ACL injuries were recorded (9.7%). A total of 376 patients (71.5%) achieved an LSI of ≥90% (94% ± 12) in knee flexion strength and were classified into the reference group. Within this group, 43 second ACL injuries (11%) were reported in the 24 months following ACL reconstruction. The LSI 80%–89.9% group comprised 101 patients, with 4 ipsilateral second ACL injuries (4.0%), and the LSI < 80% group included 49 patients, with 4 ipsilateral second ACL injuries (8.2%). Table 1 presents the groups divided by knee flexion strength LSI at the time of RTS. Patients who sustained a second ACL injury within 24 months after ACL reconstruction were notably younger at the time of surgery (20 ± 5 years vs. 22 ± 6 years, *p* = 0.005) compared to patients who did not sustain a second ACL injury. Additionally, patients who sustained a second ACL injury had significantly shorter time to RTS compared to patients who did not sustain a second ACL injury (8.6 ± 3.0 months vs. 9.9 ± 4.5 months) respectively, *p* = 0.04). Appendix 1 presents the groups categorised by patients who sustained a second ACL injury versus patients with no second ACL injury.

**Table 1:** Demographic data for patients divided into groups based on knee flexion strength LSI at the time of return to sport.

	Total	Knee flexion LSI < 80%	Knee flexion LSI 80%–89.9%	Knee flexion LSI ≥ 90%	Effect size	*p* value
Subjects	526	49 (9.3%)	101 (19.2%)	376 (71.5%)		
Female, n (%)	251 (47.7%)	17 (6.8%)	47 (18.7%)	187 (74.5%)		0.700
Male, n (%)	275 (52.3%)	32 (11.7%)	54 (19.6%)	189 (68.7%)		
Age at ACLR (years)	22.3 ± 6	24.4 ± 7	22.7 ± 6	22.0 ± 6	0.036	0.010
Height (cm)	175 ± 9	176 ± 10	175 ± 8	175 ± 10	0.0	0.930
Weight (kg)	72 ± 12	76 ± 12	73 ± 11	72 ± 12	0.012	0.049
BMI (kg/m^2^)	23 ± 2	24 ± 3	24 ± 3	23 ± 3	0.028	0.005
Time from injury to ACLR (months)	7.2 ± 11	10.1 ± 22	8.2 ± 13	6.1 ± 8	0.018	0.200
Time from ACLR to RTS (months)	9.6 ± 4	6.9 ± 5	9.3 ± 5	10.1 ± 4	0.053	<0.001
Pre‐injury Tegner level, *n* (%)						
Level 6	25 (4.8%)	2 (4.1%)	4 (4.0%)	19 (5.0%)		
Level 7	68 (12.9%)	6 (12.2%)	11 (10.9%)	51 (13.6%)		
Level 8	148 (28.1%)	13 (26.5%)	28 (27.7%)	107 (28.5%)		
Level 9	182 (34.6%)	19 (38.8%)	38 (37.6%)	125 (33.2%)		
Level 10	103 (19.6%)	9 (18.4%)	20 (19.8%)	74 (19.7%)	0.038	0.500
Knee extension ≥ 90%						
Yes, *n* (%)	376 (71.5%)	20 (40.8%)	65 (64.4%)	291 (77.4%)	0.245	<0.001
No, *n* (%)	150 (28.5%)	29 (59.2%)	36 (35.6%)	85 (22.6%)		
Hop tests ≥ 90%						
Yes, *n* (%)	240 (45.6%)	13 (48.1%)	38 (48.7%)	189 (56.4%)	0.067	0.210
No, *n* (%)	200 (38.0%)	14 (51.9%)	40 (51.3%)	146 (43.6%)		
Missing, *n*	86	22	23	41		
Second ACL injury						
Yes, *n* (%)	51 (9.7%)	4 (8.2%)	4 (4.0%)	43 (11.4%)	0.100	0.110
No, *n* (%)	475 (90.3%)	45 (91.8%)	97 (96.0%)	333 (88.6%)		
Ipsilateral	35 (68.6%)	4 (100.0%)	4 (100.0%)	27 (62.8%)	0.292	0.070
Contralateral	16 (31.4%)	0	0	16 (37.2%)		

*Note*: Values are presented as mean ± SD for continuous variables and count (percentage) for categorical variables. Effect sizes for continuous variables are reported as eta squared (*η*²) from ANOVA, and for categorical variables as Cramér's V from chi‐square tests.

Abbreviations: ACL, anterior cruciate ligament; ACLR, anterior cruciate ligament reconstruction; ANOVA, analysis of variance; BMI, Body Mass Index; cm, centimeters; kg, kilograms; LSI, limb symmetry index; m^2^, square metre; *n*, number; RTS, return to sport; Tegner, Tegner Activity Scale.

**Figure 2:**
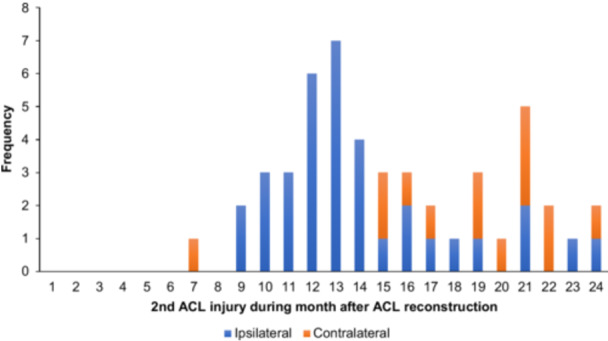
Frequency of a second anterior cruciate ligament (ACL) injury per month after ACL reconstruction.

**Figure 3:**
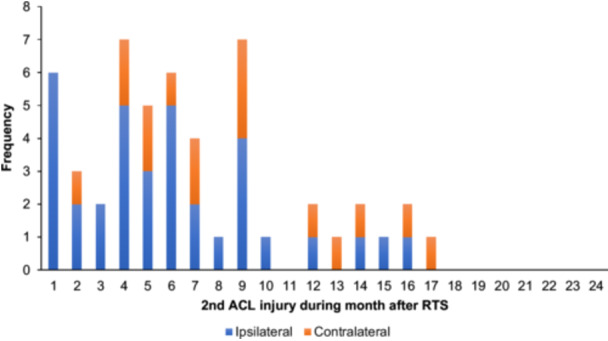
Frequency of a second anterior cruciate ligament (ACL) injury per month after return to sport (RTS).

No contralateral ACL injuries were observed in the groups with LSI < 80% and LSI 80%–89.9%. However, 16 out of the 43 s ACL injuries in the LSI ≥ 90% group occurred in the contralateral knee. The timing of a second ACL injury, divided by ipsilateral or contralateral, in relation to ACL reconstruction and RTS, are illustrated in Figures 2 and 3.

**Figure 4:**
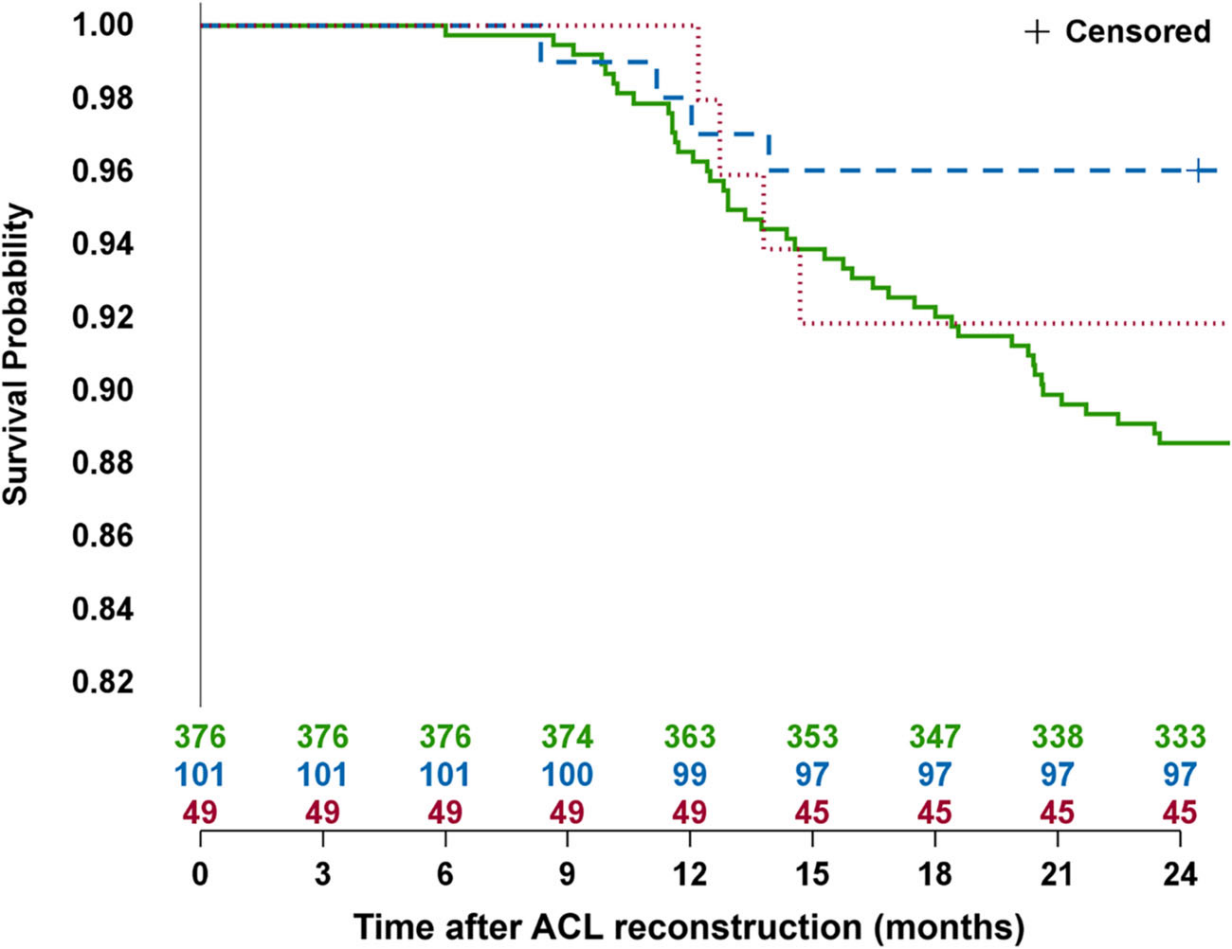
Kaplan–Meier Curve: Second ACL injury following ACL reconstruction analysed between knee flexion strength LSI groups. 1 (green). Knee flexion LSI ≥ 90%. 2 (blue). Knee flexion LSI 80‐89.9%. 3 (red). Knee flexion LSI < 80%. ACL, anterior cruciate ligament; LSI, Limb Symmetry Index.

Patients with persistent knee flexion strength asymmetry (LSI < 80% or LSI 80%–89.9%) at RTS did not show an increased rate of a second ACL injury. The LSI 80%–89.9% group had a HR of 0.34 (CI: 0.12–0.94) and the LSI < 80% group a HR of 0.70 (CI: 0.25–1.97) compared to the reference group (LSI ≥ 90%). No significant difference in the rate of a second ACL injury was observed when adjusted for symmetry in both knee extension and hop tests (LSI ≥ 90%). Table 2 presents the HRs, which indicate the hazard of a second ACL injury for different knee flexion strength LSI groups. Figure 4 displays the Kaplan–Meier curve, illustrating the survival probability (no second ACL injury) over time for the different LSI groups.

**Table 2:** Cox hazard ratios for a second ACL injury.

		Univariable HR of a second ACL injury		Adjusted HR of a second ACL injury*	
		Knee flexion LSI 80%‐89.9%	Knee flexion LSI < 80%	Knee flexion LSI 80%‐89.9%	Knee flexion LSI < 80%
*Reference*	Knee flexion ≥ 90%	HR 0.34 CI: 0.12–0.94 *p* = 0.038	HR 0.70 CI: 0.25–1.97 *p* = 0.50	HR 0.37 CI: 0.13–1.02 *p* = 0.054	HR: 0.95 CI: 0.37–2.39 *p* = 0.90
	Knee flexion 80‐89.9%		HR 2.09 CI: 0.52–8.33 *p* = 0.30		HR 2.59 CI: 0.70–9.64 *p* = 0.71

* Multivariable analysis adjusted for symmetrical knee extension and hop performance (LSI ≥ 90%).

Abbreviations: ACL, anterior cruciate ligament; CI, confidence interval; HR, hazard ratio, LSI, Limb Symmetry Index.

## DISCUSSION

The main finding of this observational cohort study was that a knee flexion strength deficit (LSI < 90%) at the time of RTS was not found to be associated with an elevated rate of a second ACL injury during 2 years after RTS. A total of 150 patients did not attain the recommended knee flexion strength symmetry (LSI ≥ 90%) yet resumed sports participation without a significantly higher rate of second ACL injury within 2 years after reconstruction, compared to the group who met the recommended LSI threshold. This finding was irrespective of whether patients achieved symmetrical knee extension and hop performance at the same test occasion.

### The significance of knee flexion strength

Although a second ACL injury is a result from various interacting factors, the emphasis on hamstring function and strength have remained consistent due to the hamstrings' crucial biomechanical role in knee joint stability. The medial hamstring is considered important to control knee valgus moments and anterior tibial translation during rapid movements in sport, such as cutting and sidestepping [6]. To restore symmetrical strength in knee flexion (LSI ≥ 90%) after HT autograft harvest for ACL reconstruction is therefore considered a benchmark for a successful rehabilitation outcome [30]. However, a systematic review by Högberg et al. [23] revealed that recovery of knee flexion strength may be delayed up to 2 years after ACL reconstruction, with significant residual impairments persistent beyond this timeframe [32]. In our study, the majority of patients (71.5%) achieved the recommended knee flexion symmetry, with a mean LSI of 94% ± 12 at the time of RTS (10.1 ± 4 months). While individual recovery varies, previous research consistently shows that most individuals achieve an LSI above 90% at the time of return to sport [29, 45]. However, it is important to recognise that a substantial proportion of patients still present with asymmetry, as seen in our cohort, where 28.5% had knee flexion strength asymmetry, and 9.3% demonstrated an LSI < 80%, without an increased occurrence of second ACL injuries.

In addition, to the interlimb strength symmetry, the value of a balanced hamstring and quadriceps strength for sufficient co‐activation and dynamic knee‐joint stability is acknowledged, however, the importance of the optimal strength relationship to reduce risk of second ACL injury remains uncertain [25, 34]. To evaluate the significance of concurrent knee extension strength and hop performance when assessing knee flexion strength, we performed a Cox proportional hazard analysis adjusted for knee extension and hop performance symmetry. However, this analysis did not demonstrate any additional impact of symmetrical muscle function on the rate of a second ACL injury for the LSI 80%–89.9% and LSI < 80% groups (HR 0.37 and 0.96, respectively), indicating the hazard of a second ACL injury was lower, although not significant, in these groups compared to the reference group (LSI ≥ 90%). Considering this, it is notable that the LSI < 80% group also exhibited greater knee extension strength asymmetry, with 59.2% of patients not achieving symmetrical strength (LSI ≥ 90%), compared to 22.6% of patients in the LSI ≥ 90% group. This suggests a broader impairment in overall muscle function recovery beyond hamstring strength. However, the lack of information on each individual's specific rehabilitation programme, along with the earlier reported RTS in this group, underscores the need to consider how rehabilitation duration and recovery goals influence muscle function outcomes [39].

### Interpretation of the LSI

When employing LSI cut‐off values and specific benchmarks, such as LSI ≥ 90%, to evaluate a patient's readiness to RTS with minimal risk of a second ACL injury, it is crucial to recognise the sensitivity of these LSI ratios. Even small changes in absolute strength values, whether in the reconstructed or non‐injured limb, can significantly shift the reported ratio, and place the patient on either the ‘pass’ or ‘fail’ side of the benchmark. This becomes particularly relevant when dealing with lower absolute values in Nm (e.g., knee flexion values vs. extension, paediatric patients or females), as even minor variations between strength assessments can result in large fluctuations in the reported LSI. Consequently, LSI and specific benchmarks for ‘pass’ or ‘fail’ should always be applied with caution and interpreted within the relevant context [52]. This need for caution is reinforced by critiques of the LSI's validity in predicting a second ACL injury [51]. For instance, LSI evaluations may overestimate muscle function recovery due to bilateral strength deficits postoperatively, where the contralateral non‐injured limb shows a decline compared to strength levels before ACL reconstruction. This, in turn may also affect the risk for a second ACL injury at RTS [52]. As an alternative, the individual's pre‐operative strength values or normative values for a reference group (sex, age and level of sport) have been endorsed as more reliable benchmarks for assessing recovery of muscle strength [42, 49]. In our study, we focused exclusively on LSI levels and did not analyse relative strength (torque to body weight) or compare results to normative values based on sex, sport, or age. However, with regard to the phenomenon of bilateral strength deficit and the similar occurrence rates of contralateral ACL injuries and ipsilateral graft re‐ruptures, we included both types of second ACL injuries. We recorded 35 (68.6%) ipsilateral graft re‐ruptures and 16 (31.4%) contralateral ACL injuries, with a slightly higher proportion of contralateral ACL injuries in females as previously reported [40]. Notably, 79.3% of ACL injuries in the first 6 months after RTS were ipsilateral graft re‐ruptures while for the remaining time spectrum 42.9% were contralateral ACL injuries. All 16 contralateral ACL injuries occurred in the LSI ≥ 90% group. The absence of ACL injuries in the other LSI groups limited our ability to conduct a comprehensive comparison of ipsilateral and contralateral injuries across the different LSI groups. Whether this absence of contralateral injuries in the LSI < 80% and LSI 80–89.9% groups suggests that a second ACL injury is more likely to occur ipsilaterally, due to deficits in muscle function in the reconstructed knee, remains speculative based on the current data. Conversely, it is possible that for the group who achieved LSI ≥ 90%, the load distribution between the ACL reconstructed knee and the contralateral knee becomes more balanced, potentially leading to a more even distribution of subsequent ACL injuries [16]. Another consideration is that patients in the LSI ≥ 90% group had better perceived or actual knee function, leading to earlier and more aggressive playing behaviours at RTS, thereby subjecting the contralateral limb to greater cumulative loading and increased injury risk. The finding underscores the limitations of relying solely on LSI thresholds and emphasises the need to incorporate absolute strength benchmarks and exposure monitoring in RTS assessments.

### Testing knee flexion strength

This study used the seated isokinetic knee flexion strength test in concentric mode with an angular velocity of 90°/second for analysis. The Biodex dynamometer demonstrates good instrumental validity [14] (intraclass correlation coefficient (ICC) = 0.99–1.00) and test‐retest reliability [15] (ICC = 0.95) when measuring peak torque in knee extension and knee flexion. The seated isokinetic knee flexion is also reported as the most common test for assessment of knee flexor strength after ACL reconstruction [23]. However, given the similar rates of second ACL injury between the reference group (LSI ≥ 90%) and the group with the most pronounced knee flexion strength deficit (LSI < 80%), our findings raise the question of whether the seated isokinetic test of knee flexion strength is a valid tool to assess the hamstrings ability to contribute to dynamic stabilisation of the knee‐joint after ACL reconstruction and ultimately to screen for a second ACL injury risk [22, 27]. Patients with HT autografts are known to display muscle atrophy and proximal retraction of the ST muscle with a more distinctive strength deficit in the inner range of knee flexion ( > 75°) and a compensatory hypertrophy of the lateral hamstring (biceps femoris), leading to a shifted internal to external knee rotation strength ratio [38]. Despite morphological alterations after HT harvesting, full restoration of symmetrical knee flexion strength in the seated position (LSI ≥ 90%) has been reported more often and sooner after ACL reconstruction compared to knee extension strength and vertical hop performance [11, 18]. In addition to the criticism of the seated test position, LSI strength evaluations after ACL reconstruction may be questioned for relying solely on the peak torque values without considering the torque‐to‐joint angle relationship. Specifically, the oversight of knee flexion strength close to full knee extension angles, where the ACL is reported to rupture during common injury situations such as cutting or landing actions in sports [55], has been highlighted as a neglected aspect of the hamstrings' role in dynamically stabilising the knee [44].

## LIMITATIONS

This study included 526 patients aged 15–40 years, among whom 51 experienced a second ACL injury (9.7%), which indicates a lower incidence compared to similar cohorts in previous research [4, 41]. While the use of the Project ACL registry provided access to a sizable cohort, the small number of second ACL injuries limits the ability to establish a robust association between knee flexion LSI and a second ACL injury. Future investigations could benefit from a larger cohort to enhance subgroup analyses by sex, age or sports level, especially given the observed differences in age at ACL reconstruction and time to RTS across LSI groups, which are established risk factors for a second ACL injury [41, 50]. Additionally, there was a significant imbalance in group sizes, with 71.5% of participants in the LSI ≥ 90% group and only 9.3% in the LSI < 80% group, which potentially limited the statistical power of LSI group comparisons. The low numbers in the LSI < 80% group reduce the ability to detect significant differences and increase the risk of Type II errors, where important differences or associations may be overlooked due to insufficient sample size. A further limitation is the exclusion of 519 patients whose RTS status did not meet the inclusion criteria and baseline characteristics were unavailable, preventing direct comparisons and potentially introducing selection bias.

We defined RTS using a patient‐reported Tegner level of ≥6. However, this criterion does not ensure a return to the pre‐injury activity level. Moreover, a reported Tegner level of ≥ 6 does not distinguish between the risk for a second ACL injury among patients who RTS earlier at a lower Tegner level compared to a later RTS at pre‐injury Tegner level. For example, the LSI < 80% group reported RTS earlier (6.9 months) than the LSI ≥ 90% group (10.1 months), yet experienced fewer second ACL injuries. Although the Project ACL registry does not track rehabilitation specifics, future studies should consider to incorporate measures such as rehabilitation duration or estimated number of rehabilitation sessions to better contextualise recovery trajectories and their influence on second ACL injury risk. Additionally, more precise methods are needed to evaluate the timing of RTS and to adequately quantify athletic exposure after RTS, including training volume, intensity, and the number of training sessions, matches, and competitions.

Additionally, the chosen method, to study knee flexion strength deficits at the time of RTS, can be questioned as no further assessments of the LSI of knee flexion strength were conducted at follow‐ups after reported RTS. It is possible that a patient with a knee flexion strength deficit at the time of RTS may regain strength later during sports participation, as symmetrical strength restoration has been reported to take up to 2 years after HT graft harvesting [23]. To better evaluate the significance of an LSI deficit in knee flexion strength on the risk of a second ACL injury, knee flexion strength should be monitored continuously during the RTS phase of rehabilitation and the first year of sports participation.

Finally, it is important to acknowledge that second ACL injuries are multifactorial. This analysis did not account for other potential factors, such as anatomical characteristics [1, 36], meniscal or cartilage lesions [10] or surgical preference and techniques in ACL reconstruction [17, 33, 43].

## CONCLUSION

A persistent knee flexion strength asymmetry (LSI < 90%) at the time of RTS was not associated with an increased rate of a second ACL injury within 2 years after ACL reconstruction. This outcome was consistent regardless of whether symmetrical knee extension and hop performance were achieved at the same test occasion.

## AUTHOR CONTRIBUTIONS

All authors contributed to the study conception and design. Material preparation, data collection and analysis were performed by author Axel Sundberg. The first draft of the manuscript was written by Axel Sundberg and all authors commented on previous versions of the manuscript. The manuscript was supervised by authors Kristian Samuelsson and Eric Hamrin Senorski. All authors read and approved the final manuscript.

## CONFLICT OF INTEREST STATEMENT

Author Kristian Samuelsson is a board member at Getinge AB.

## ETHICS STATEMENT

This study was conducted in accordance with the Declaration of Helsinki and approved by the Swedish Ethical Review Authority, registration number: 2020‐02501. All patients provided informed consent for their data to be included in the rehabilitation registry and used for research purposes. Data were anonymized before analysis to ensure patient confidentiality.

## Figures and Tables

**Figure B1:**
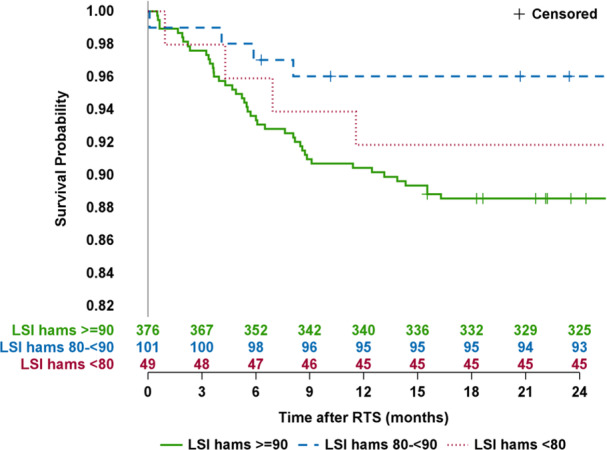
Kaplan–Meier Curve: Second ACL injury following reported time of RTS analysed between knee flexion strength LSI groups. ACL, anterior cruciate ligament; LSI, Limb Symmetry Index; RTS, return to sport.

## Data Availability

Data are available upon reasonable request. Data can be obtained on request due to privacy reasons and ethical statements.

## Appendix 1

See Table A1.

**Table A1:** Demographic data for patients based on second ACL injury occurrence or not.

	Total	No second ACL injury	Second ACL injury	*p* value
*n* (%)	526	475 (90.3%)	51 (9.7%)	
Female	251 (48%)	226 (47.6%)	25 (49.0%)	0.88
Male	275 (52%)	249 (52.4%)	26 (51.0%)	
Age at ACLR (years)	22.2 ± 6	22.4 ± 6	20.3 ± 5	0.005
Height (cm)	175.1 ± 9	175.3 ± 9	174.4 ± 8	0.56
Weight (kg)	72.2 ± 12	72.4 ± 12	73.2 ± 13	0.61
BMI (kg/m^2^)	23.1 ± 2	23.1 ± 2	23.2 ± 3	0.20
Time from injury to ACLR (months)	7.0 ± 11.4	7.3 ± 12.0	4.5 ± 3.9	0.09
Time from ACLR to RTS (months)	9.8 ± 4.4	9.9 ± 4.5	8.6 ± 3.0	0.04
Pre‐injury Tegner level, *n* (%)				
Level 6	25 (4.8%)	25 (5.3%)	0	0.20
Level 7	68 (12.9%)	64 (13.5%)	4 (7.8%)	
Level 8	148 (28.1%)	130 (27.4%)	18 (35.3%)	
Level 9	182 (34.6%)	166 (34.9%)	16 (31.4%)	
Level 10	103 (19.6%)	90 (18.9%)	13 (25.5%)	
Total	526 (100%)	475 (100%)	51 (100%)	
Second ACL injury, *n* (%)				
Ipsilateral			35 (69%)	0.07
Contralateral			16 (31%)	

*Note*: Values are presented as mean ± SD for continuous variables and count (percentage) for categorical variables.

Abbreviations: ACL, anterior cruciate ligament; ACLR, anterior cruciate ligament reconstruction; BMI, body mass index; cm, centimeters; kg, kilograms; m^2^, square metre; *n*, number; RTS, return to sport; SD, standard deviation; Tegner, Tegner Activity Scale.

## Appendix 2

See Figure B1.
